# Realistic Reference for Evaluation of Vehicle Safety Focusing on Pedestrian Head Protection Observed From Kinematic Reconstruction of Real-World Collisions

**DOI:** 10.3389/fbioe.2021.768994

**Published:** 2021-12-21

**Authors:** Guibing Li, Jinming Liu, Kui Li, Hui Zhao, Liangliang Shi, Shuai Zhang, Jin Nie

**Affiliations:** ^1^ School of Mechanical Engineering, Hunan University of Science and Technology, Xiangtan, China; ^2^ Chongqing Key Laboratory of Vehicle Crash/Bio-Impact and Traffic Safety, Institute for Traffic Medicine, Daping Hospital, Army Medical University, Chongqing, China; ^3^ China Automotive Engineering Research Institute Co., Ltd., Chongqing, China; ^4^ The Fifth Institute of Army Academy, Wuxi, China; ^5^ Loudi Vocational and Technical College, Loudi, China

**Keywords:** pedestrian head-to-vehicle contact, boundary condition, injury risk, kinematic reconstruction, real-world accidents

## Abstract

Head-to-vehicle contact boundary condition and criteria and corresponding thresholds of head injuries are crucial in evaluation of vehicle safety performance for pedestrian protection, which need a constantly updated understanding of pedestrian head kinematic response and injury risk in real-world collisions. Thus, the purpose of the current study is to investigate the characteristics of pedestrian head-to-vehicle contact boundary condition and pedestrian AIS3+ (Abbreviated Injury Scale) head injury risk as functions of kinematic-based criteria, including HIC (Head Injury Criterion), HIP (Head Impact Power), GAMBIT (Generalized Acceleration Model for Brain Injury Threshold), RIC (Rotational Injury Criterion), and BrIC (Brain Injury Criteria), in real-world collisions. To achieve this, 57 vehicle-to-pedestrian collision cases were employed, and a multi-body modeling approach was applied to reconstruct pedestrian kinematics in these real-world collisions. The results show that head-to-windscreen contacts are dominant in pedestrian collisions of the analysis sample and that head WAD (Wrap Around Distance) floats from 1.5 to 2.3 m, with a mean value of 1.84 m; 80% of cases have a head linear contact velocity below 45 km/h or an angular contact velocity less than 40 rad/s; pedestrian head linear contact velocity is on average 83 ± 23% of the vehicle impact velocity, while the head angular contact velocity (in rad/s) is on average 75 ± 25% of the vehicle impact velocity in km/h; 77% of cases have a head contact time in the range 50–140 ms, and negative and positive linear correlations are observed for the relationships between pedestrian head contact time and WAD/height ratio and vehicle impact velocity, respectively; 70% of cases have a head contact angle floating from 40° to 70°, with an average value of 53°; the pedestrian head contact angles on windscreens (average = 48°) are significantly lower than those on bonnets (average = 60°); the predicted thresholds of HIC, HIP, GAMBIT, RIC, BrIC2011, and BrIC2013 for a 50% probability of AIS3+ head injury risk are 1,300, 60 kW, 0.74, 1,470 × 10^4^, 0.56, and 0.57, respectively. The findings of the current work could provide realistic reference for evaluation of vehicle safety performance focusing on pedestrian protection.

## Background

In vehicle-to-pedestrian crashes, injuries to the head account for more than 30% of all AIS2+ (Abbreviated Injury Scale) injuries to pedestrians (Mizuno, 2005), which are also the main causes of pedestrian death (Yang, 2005; Simms and Wood, 2009; Schmitt et al., 2010). Hence, pedestrian head protection is one of the most important assessments of vehicle safety performance in regulations, such as the New Car Assessment Programs (NCAPs) in different countries and regions (C-NCAP, 2020; Euro-NCAP, 2020; J-NCAP, 2014), which have significantly improved current vehicle safety design (Li et al., 2018 and 2019). In NCAPs, subsystem tests using isolated impactors with the consideration of head-to-bonnet/windshield area impacts are employed, where the definition of the impact boundary condition and the criterion and corresponding thresholds for head injuries are crucial since these have a significant influence on vehicle safety (pedestrian injury risk) rating (C-NCAP, 2020; Euro-NCAP, 2020). Thus, a good understanding of pedestrian head-to-vehicle contact boundary condition and head injury risk in real-world collisions can help improve the effectiveness of head impactor subsystem tests in vehicle safety evaluation.

In subsystem tests, the head-to-vehicle contact boundary condition is defined as a constant linear impact speed at a certain angle (relative to the horizontal plane) to the head impactor; e.g., the C-NCAP and C-IASI use a linear head impact speed of 40 km/h at 65° for adult head impactor tests (C-IASI, 2017; C-NCAP, 2020), which is the same as defined in the Euro-NCAP (2020). However, the head-to-vehicle contact boundary condition defined in the current C-NCAP and C-IASI may not suit the situation in China due to the differences in characteristics of traffic accidents between China and Europe as reported in previous studies (Chen et al., 2009; Yang and Otte, 2007). Moreover, the rotational kinematics of pedestrian head-to-vehicle contact is not considered in the current NCAPs, which is an important factor affecting head injury risk (Gennarelli et al., 1971; Rowson et al., 2011; Takhounts et al., 2011 and 2013). On the other hand, NCAPs in different countries and regions currently use the Head Injury Criterion (HIC) and relative thresholds to assess pedestrian head injury risk (C-NCAP, 2020; Euro NCAP, 2020). However, the HIC is controversial for predicting brain injury risk as it only considers head linear acceleration while some brain injuries are usually caused by rotational motion or combining linear motion (Schmitt et al., 2010; Simms and Wood, 2009; Yang, 2005). Given this, some other kinematic-based criteria were proposed, considering rotational response only or combining linear and rotational response for Traumatic Brain Injury (TBI), such as the Head Impact Power (HIP) (Newman et al., 2000), Generalized Acceleration Model for Brain Injury Threshold (GAMBIT) (Newman, 1986), Rotational Injury Criterion (RIC) (Kimpara and Iwamoto, 2012), and Brain Injury Criteria (BrIC) (Takhounts et al., 2011 and 2013). Accordingly, the thresholds or risk curves of human head injuries at different AIS levels were also proposed for these criteria, based on data from physical impact tests, reconstruction of real-world accidents (football players, motorcyclists, pedestrians, etc.) using isolated Anthropomorphic Test Devices (ATDs) and/or numerical human body models of head (Kimpara and Iwamoto, 2012; Marjoux et al., 2008; Newman, 1986; Takhounts et al., 2011 and 2013). However, few studies have focused on developing injury risk curves from reconstruction of real-world pedestrian crashes at the full-body level, covering all the above-mentioned kinematic-based criteria, where the influence of restriction from the torso and lower body on pedestrian head dynamic response and the cumulative head kinematics in the whole impact can be considered.

Therefore, the purpose of the current study is to investigate the characteristics of pedestrian head-to-vehicle contact boundary condition and to develop pedestrian head injury risk curves *via* kinematic reconstruction of real-world collisions. To achieve this, 57 vehicle-to-pedestrian collision cases were employed, and a multi-body modeling approach was applied to reconstruct pedestrian kinematics in these real-world collisions. Based on the reconstruction data, the characteristics of pedestrian head-to-vehicle contact boundary condition were then analyzed, and the pedestrian head injury risk curves as functions of kinematic-based criteria (HIC, HIP, GAMBIT, RIC, and BrIC) were built. The findings of the current work can provide realistic reference for evaluation of vehicle safety focusing on pedestrian head protection.

## Methods

### Collision Data

In the current work, 57 vehicle-to-pedestrian collision cases were selected, where 38 cases with video data captured during 2012–2019 were from the database of the Institute for Traffic Medicine, Army Military University, China, and another 19 cases were from the database of IVAC (In-Depth Investigation of Vehicle Accidents in Changsha). These enrolled cases have met the following inclusion criteria: (1) at least one AIS1+ head injury was recorded using the AIS 2005 classification (Gennarelli and Wodzin, 2006), (2) collisions involved passenger cars, (3) pedestrians are adult and were hit only once by a vehicle’s front, (4) information on vehicle (model and year), pedestrian (gender, height, weight, and age), and injuries was recorded, and (5) the collision information (vehicle impact speed, initial contact location, etc.) can be calculated/estimated. Supplementary Appendix Table S1 shows the general information of these 57 cases, where the average height for female is 157 cm and that for male is 170 cm, which generally match the stature of the Chinese population. In this sample, 39 cases are sedan crashes, while the other 18 cases are collisions with SUV (Sport Utility Vehicle) or MPV (Multi-purpose Vehicles).

### Pedestrian Head Kinematic and Injury Reconstruction

The multi-body modeling method applied by the MADYMO software (MADYMO, 2013b) was used for kinematic reconstruction of pedestrians involved in the 57 selected cases, where MADYMO pedestrian models (MADYMO, 2013a) scaled to the height and weight of the victims were employed. Similar to previous reconstruction work (Nie et al., 2014; Peng et al., 2013; Wu et al., 2021), each vehicle model was developed based on the geometric data measured from the blueprint or real car of the corresponding accident vehicle, and stiffness characteristics of the components were obtained from impactor test data reported in previous studies (Mizuno et al., 2001; Martinez et al., 2007) according to the available rating results of the vehicle model in subsystem tests.

**FIGURE 1:**
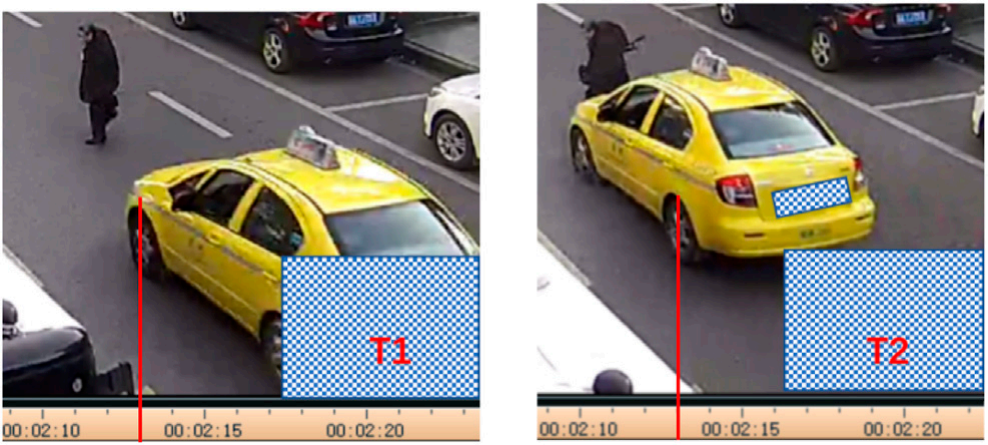
Videos captured for estimation of vehicle impact speed and pedestrian posture.

For the cases with video data, the pedestrian posture and vehicle impact speed at the instant of vehicle-to-pedestrian contact were firstly estimated from the video. Figure 1 shows a sample, where the vehicle impact speed was calculated as the result of the wheelbase (2.5 m as measured) divided by the time between the video frame T1 and T2 (number of frames passed/frame rate = 4/25 fps = 0.16 s); thus, the vehicle impact speed was 15.63 m/s (2.5 m/0.16 s = 15.63 m/s) for this case. For the cases without video data, the impact speed was estimated initially based on the data kinetic energy theorem calculation (*v* = 3.6*2 µg/L^2^: *v* is the impact speed in km/h, *µ* is the friction coefficient, *g* = 9.8 m/s^2^, and *L* is the breaking distance) and interview with the drivers.

**FIGURE 2:**
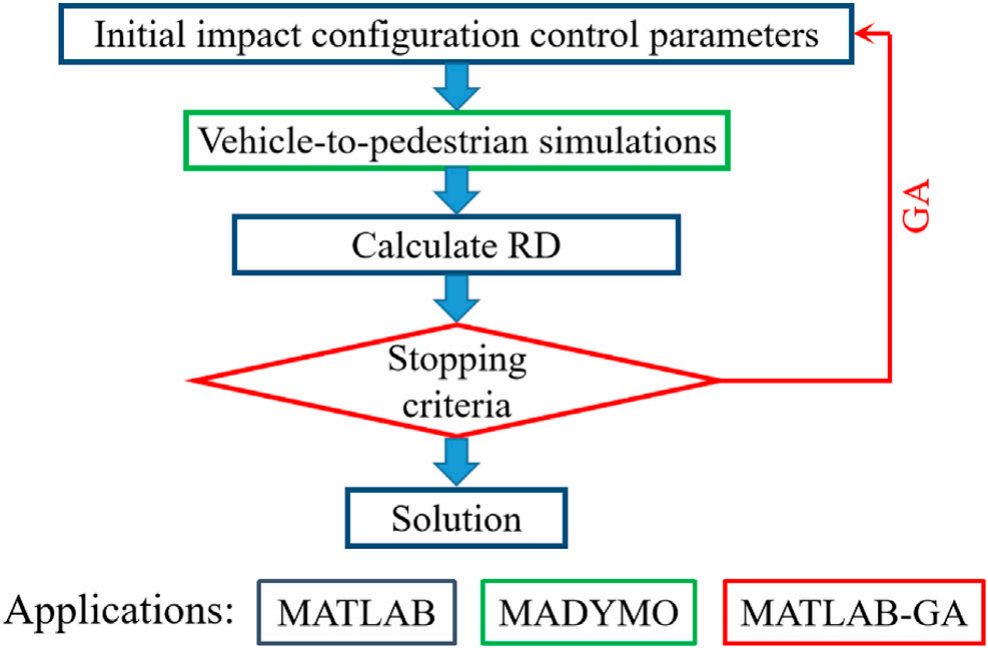
Optimization framework for kinematic reconstruction.

For each case, a basic multi-body simulation of vehicle-to-pedestrian impact was then developed based on the estimated initial impact configuration (vehicle impact speed and pedestrian initial posture and contact position). Since the initial posture (gait stance, orientation, body bending state, etc.) of pedestrian at the instant of vehicle contact is one of the important factors affecting pedestrian kinematics and injury outcome (Untaroiu et al., 2009; Elliott et al., 2012; Chen et al., 2015). For the cases with video data, we defined the initial pedestrian posture in the simulations according to that in the video, while for cases without video data, pedestrian orientation and gait stance were defined according to the accident data (orientation was recorded by clock direction, and gait stance was simply recorded as struck leg forwarding or lagging) recorded by the accident investigation team after analysis of onsite, interview, injury location, and vehicle deformation information. Then, an optimization study using the framework shown in Figure 2 was conducted to find the optimal parameters of initial impact configuration for kinematic reconstruction.

In the optimization, similar to the previous study (Li et al., 2017b; Li et al., 2020), the Genetic Algorithm (GA) defined in the MATLAB Global Optimization Box was used to find the optimal solution, and the stopping criterion was defined as the maximum of 15 generations. Similar to the study of Untaroiu et al. (2009), the fitness function was defined so as to minimize the sum of relative distance (RD) for each pedestrian contact location between prediction from the simulation and observation of the real-world case, which is calculated as follows:

$$\mathrm{RD}=\sum RD_{i}+RD_{\mathrm{final}},$$ (1)

where *RD*_*i*_ is the relative distance for each observable pedestrian contact location on the vehicle and *RD*_*final*_ is the relative distance of the final position of the pedestrian. For the cases without video data, variation ranges of ±10 km/h, ±30°, ±100 mm, and 10 different gait stances were set in the optimization study for vehicle impact speed, and pedestrian initial orientation, contact position, and gait stance, respectively.

**FIGURE 3:**
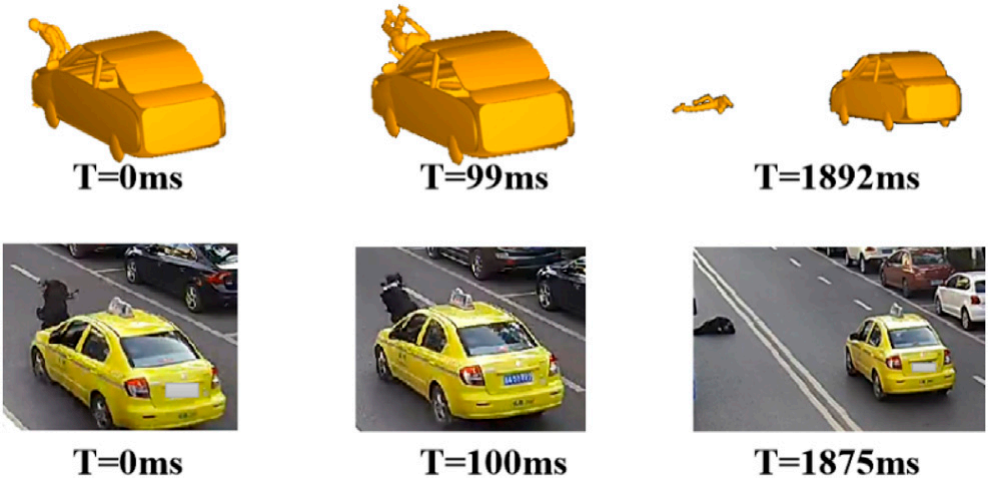
Comparison of pedestrian kinematics between reconstruction and video data.

**FIGURE 4:**
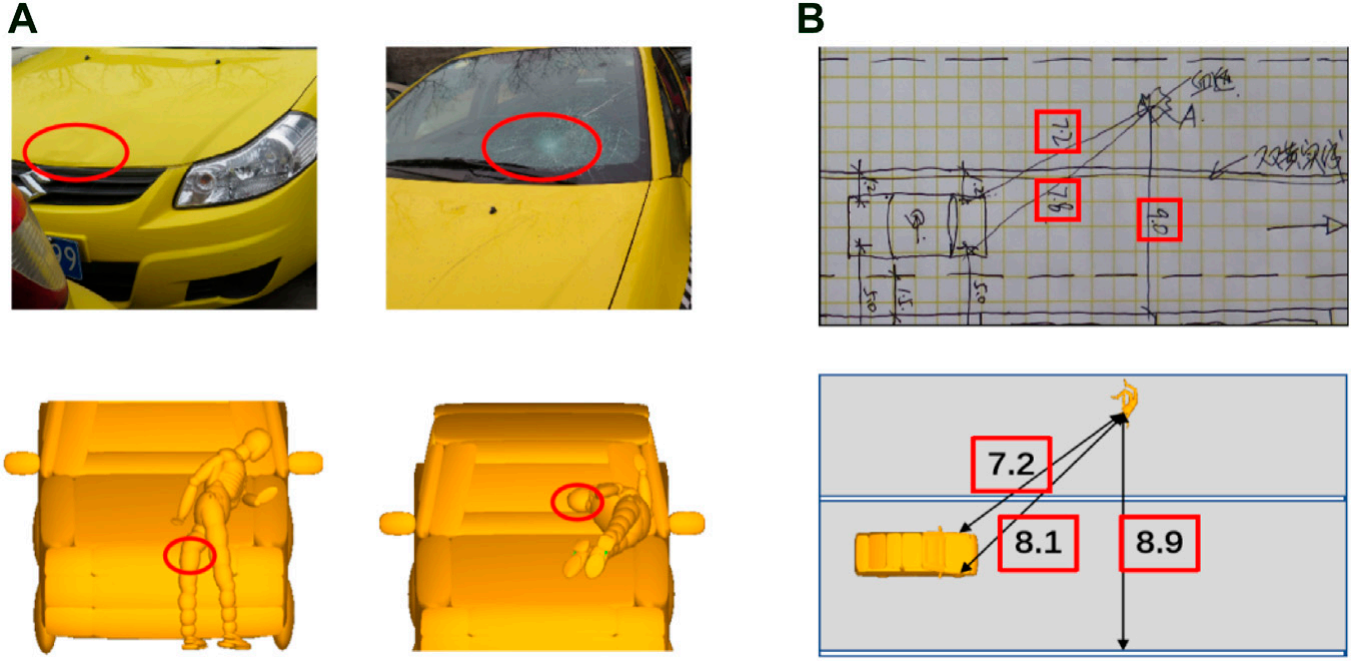
Comparison of pedestrian contact location **(** **A** **)** and final position **(** **B** **)** between reconstructions and real-world cases.

**FIGURE 5:**
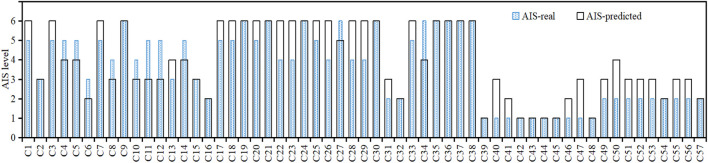
Comparison of pedestrian head injury AIS level between reconstructions and real-world cases.

Finally, a kinematic reconstruction simulation was carried out using the optimal parameters of initial impact configuration for each case. Figure 3 shows a sample of comparison between the predicted pedestrian kinematics from the reconstruction and the video data, where the predicted specific pedestrian kinematics (leg-to-vehicle contact, head-to-vehicle contact, and pedestrian-to-ground contact) and corresponding time from the reconstruction simulation can generally match the video data. Figure 4 shows a sample of comparison between the predicted pedestrian contact locations and those on the accident vehicle; again, a good match can also be observed. To evaluate the stiffness assumptions, preliminary analysis was carried out to compare the AIS level of head injuries between the reconstructions and real-world cases (see Figure 5), where the predicted AIS levels were calculated from the predicted HIC using the regression models proposed by the NHTSA (National Highway Traffic Safety Administration) with a cut line of an injury risk of greater than 50% (NHTSA, 1995). Generally, the predicted AIS level shows a good match with the real-world accident data. These indicate that pedestrian kinematic and injury reconstruction using multi-body modeling is basically plausible.

### Parameters of Pedestrian Head-to-Vehicle Contact Boundary Condition and Injury Criteria

**FIGURE 6:**
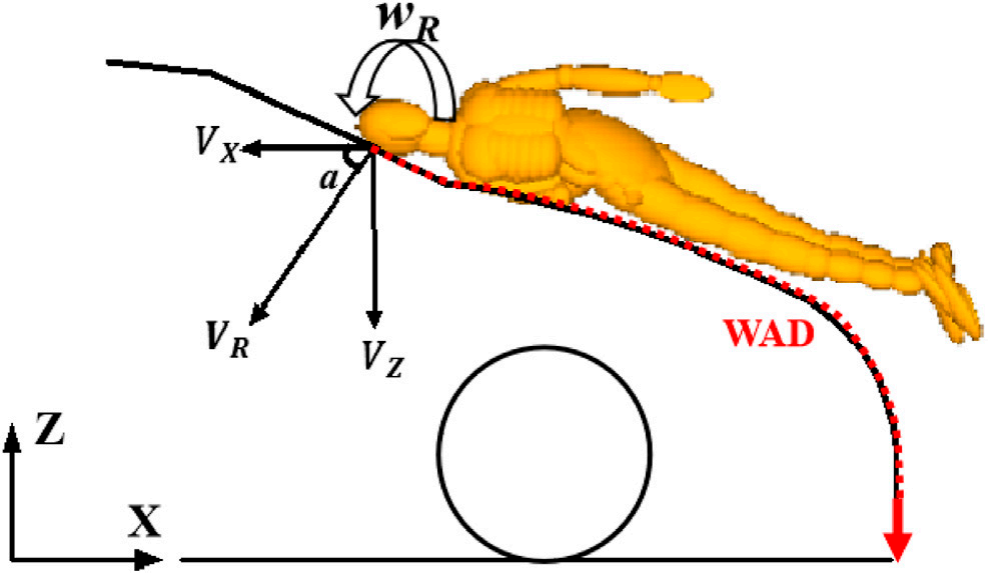
Definition for parameters of pedestrian head-to-vehicle contact boundary condition.

In the current work, pedestrian head-to-vehicle contact boundary conditions (Figure 6), including linear contact velocity (***V***_***R***_: the resultant head linear velocity relative to the vehicle at the instant of head contact), angular contact velocity (***w***_***R***_: the resultant head rotational velocity around its center of gravity at the instant of head contact), contact angle (***a***: the angle of linear contact velocity relative to the horizontal plane), WAD (Wrap Around Distance), head contact time (time period between pedestrian initial contact and the instant of head contact), were extracted from simulations of pedestrian kinematic reconstruction. The predicted head kinematics of linear and angular acceleration and velocity extracted from the 57 reconstructions was employed to calculate the kinematic-based head injury criteria of HIC, HIP, GAMBIT, RIC, and BrIC according to the formulations introduced in Supplementary Appendix Information S1.

### Statistical Analysis

Similar to previous studies (Shang et al., 2017; Li et al., 2018), the Mann–Whitney *U*-test, which is suitable for normally distributed and non-normally distributed data, was used to compare the difference in the distribution of a given parameter between two different groups (such as bonnet vs. windscreen impacts and sedan vs. SUV/MPV crashes), where the *p*-value was employed to indicate the statistical significance level of a comparison. The linear regression model was employed to analyze the relationship between two parameters with an *R* ^2^ value to assess the fitness level. The Weibull model was used to build the risk curves of serious head injury (AIS3+) as functions of the above kinematic-based head injury criteria similar to previous studies (Kerrigan et al., 2004; Takhounts et al., 2011 and 2013), where the probability of AIS3+ head injury as a function of a given criterion is given by

$$P_{\text{AIS}3+}=1-e^{-{\left(x/a\right)}^{b}},$$ (2)

where *x* is the predictor, *a* and *b* are coefficients calculated from Weibull fitting. The AUC (Area Under the Curve) value with a threshold of 0.5 was employed to evaluate the predictive capability of the Weibull models similar to a previous study (Wu et al., 2021). The AUC can be calculated by two measures, namely, false-positive rate (FPR) and true-positive rate (TPR), where FPR refers to the ratio of false-positive cases (the cases that were predicted positive but are actually negative) out of all negative cases and TPR refers to the ratio of true-positive cases (the cases that were predicted positive and are actually positive too) out of all positive cases.

## Results

### Pedestrian Head-to-Vehicle Contact Boundary Condition

**FIGURE 7:**
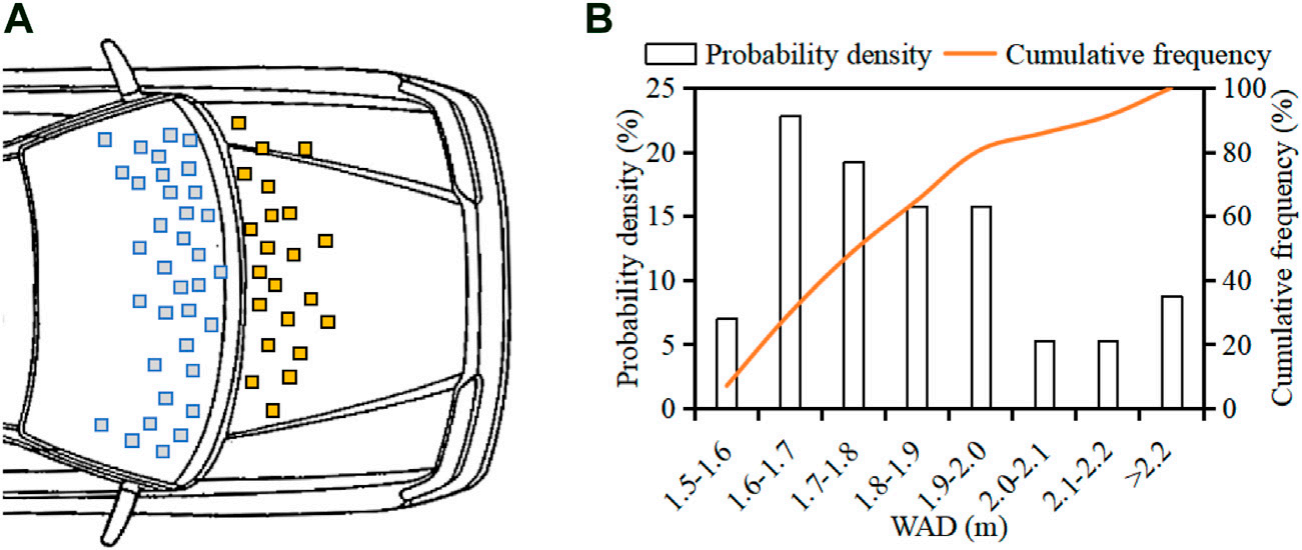
Distribution of pedestrian head-to-vehicle contact location **(** **A** **)** and WAD **(** **B** **)**.

Figure 7 shows the distribution of pedestrian head contact location and WAD. The data indicate that for 22 (38.6%) and 35 (61.4%) cases, the head contact location is on the bonnet and the windscreen, respectively. The reconstruction results show that head WAD values are in the range 1.5–2.3 m with an average of 1.84 m and that about 74% of cases have a WAD in the range 1.6–2.0 mm.

**FIGURE 8:**
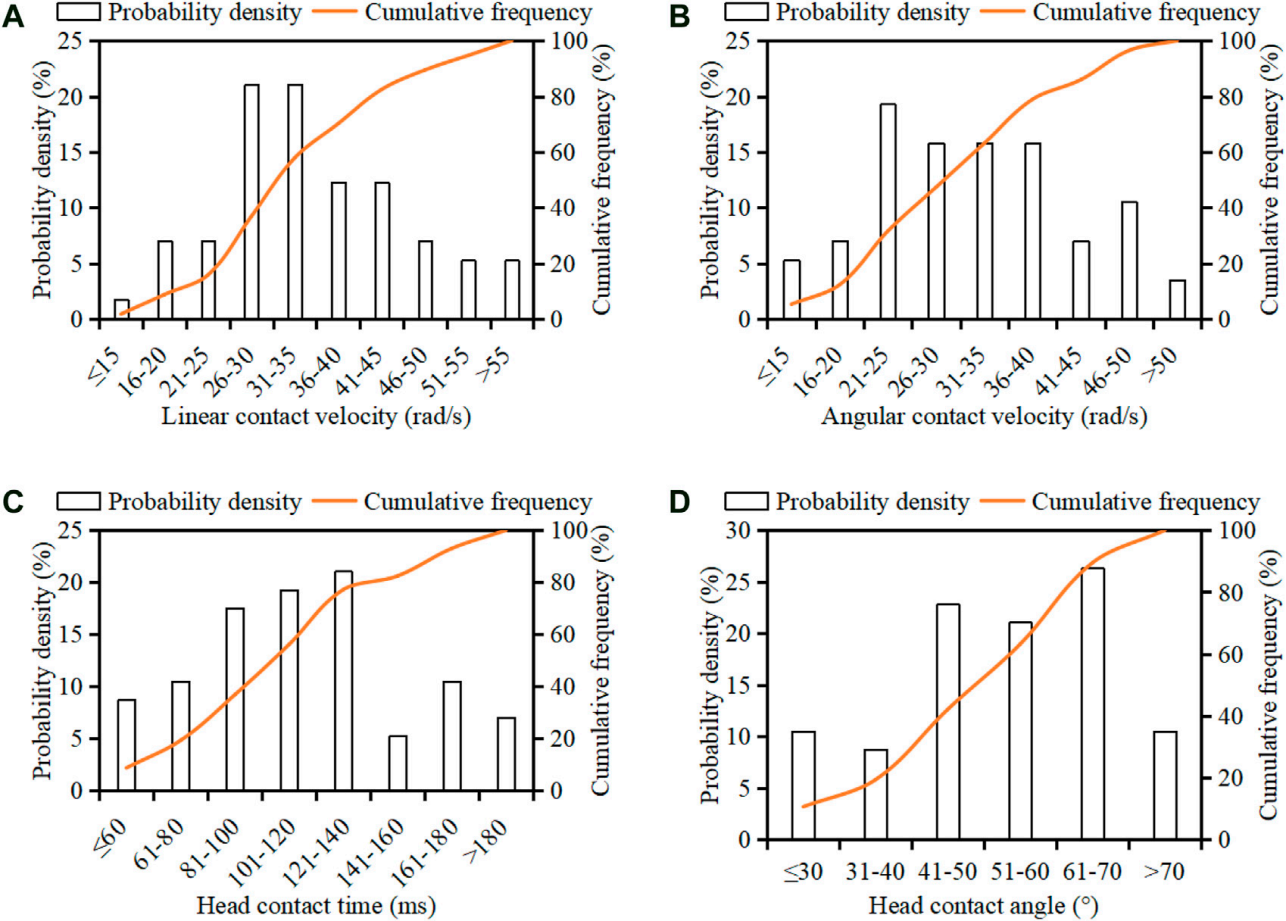
Distribution of pedestrian head linear contact velocity **(** **A** **)**, angular contact velocity **(** **B** **)**, contact time **(** **C** **)**, and contact angle **(** **D** **)**.

Figure 8 shows the distribution and cumulative frequency of pedestrian head linear contact velocity, angular contact velocity, contact time, and contact angle. Pedestrian head linear contact velocity is mainly in the range 26–45 km/h (67%), and 70% of cases have a head linear contact velocity below 40 km/h. The head angular contact velocity is mainly in the range 21–40 rad/s (67%), and 80% of cases are with a head angular contact velocity lower than 40 rad/s. More than half of the cases (58%) have a head contact time in the period 80–140 ms, and the head contact time is generally earlier than 140 ms (77%). The head contact angle is mainly in the range 40–70° (70%), with an average value of 53°.

**FIGURE 9:**
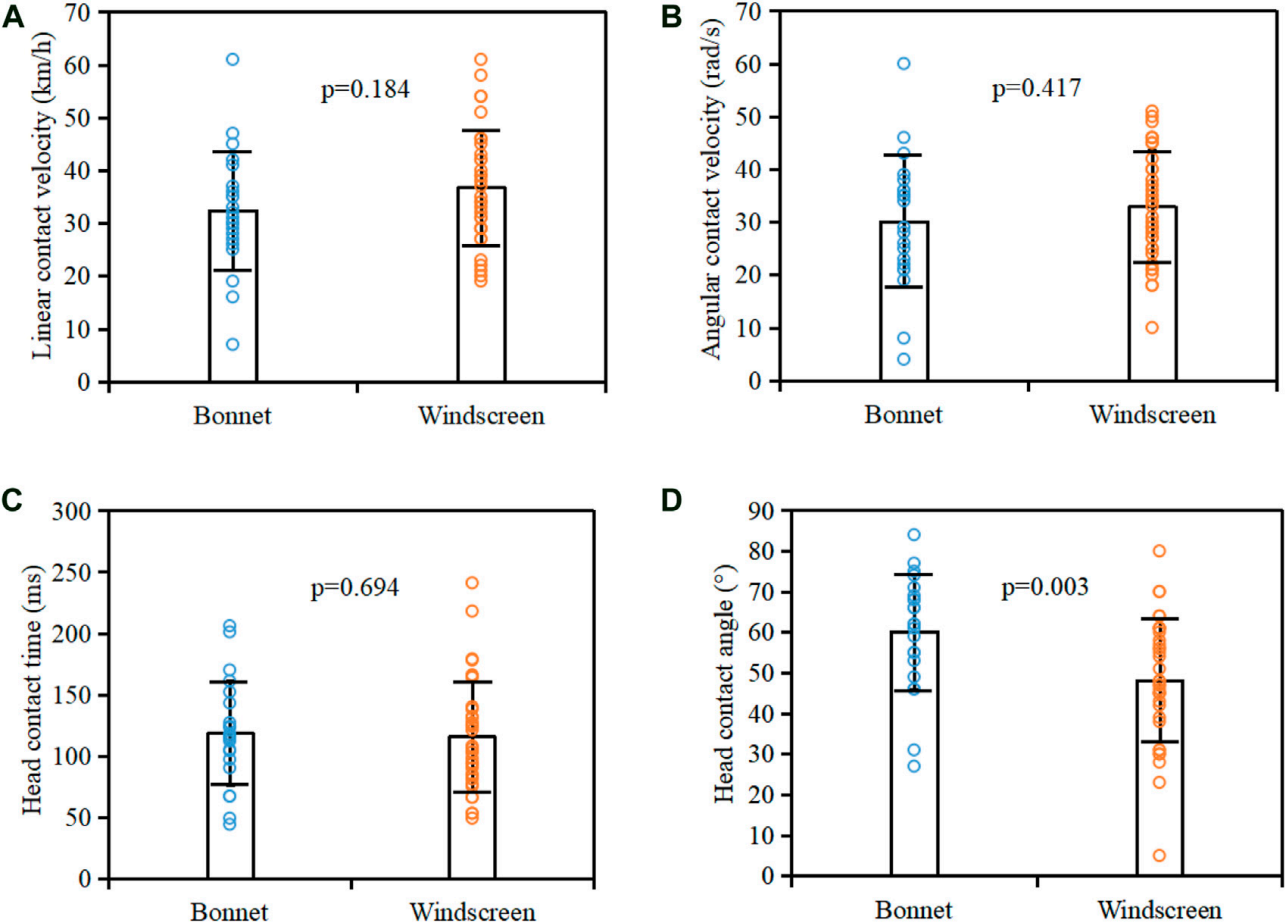
Comparison of head linear contact velocity **(** **A** **)**, angular contact velocity **(** **B** **)**, contact time **(** **C** **)**, and contact angle **(** **D** **)** between bonnet and windscreen impacts.

**FIGURE 10:**
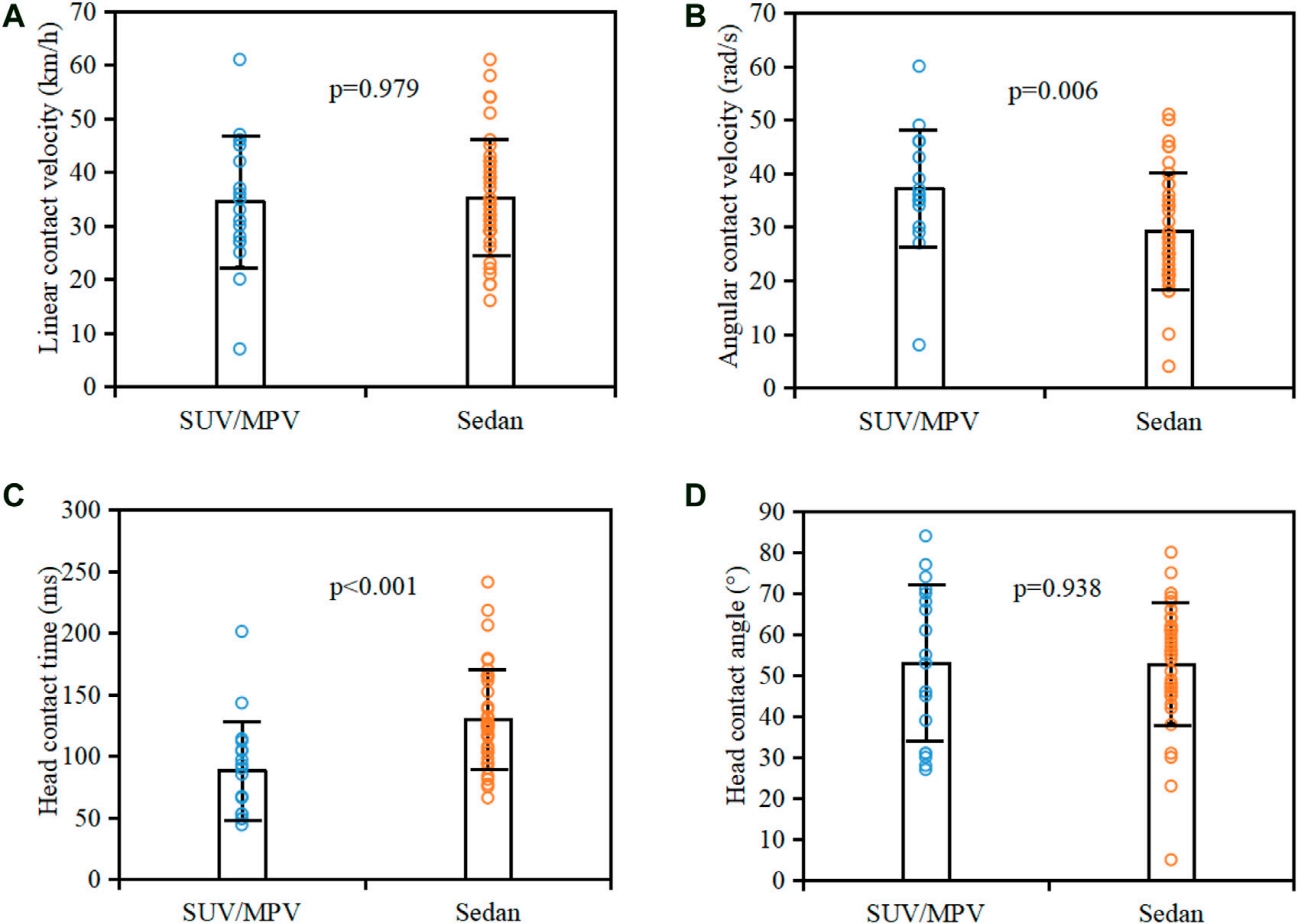
Comparison of head linear contact velocity **(** **A** **)**, angular contact velocity **(** **B** **)**, contact time **(** **C** **)**, and contact angle **(** **D** **)** between sedan and SUV/MPV crashes.

Figure 9 compares the distribution of pedestrian head linear contact velocity, angular contact velocity, contact time, and contact angle between bonnet and windscreen impacts. Generally, the head linear velocity and angular contact velocity in windscreen impacts are higher than those of bonnet impacts, but the differences are not statistically significant (*p* > 0.05). No obvious difference in head contact time was observed between bonnet and windscreen impacts, but the head contact angles for windscreen impacts are significantly bigger than those of bonnet impacts (*p* = 0.003). Figure 10 compares the distribution of pedestrian head linear contact velocity, angular contact velocity, contact time, and contact angle between sedan and SUV/MPV crashes. The SUV/MPV crashes have a significantly higher head angular contact velocity and earlier head contact time than collisions with sedans (*p* < 0.05), but no obvious differences in head linear contact velocity and contact angle were observed between sedan and SUV/MPV crashes.

**FIGURE 11:**
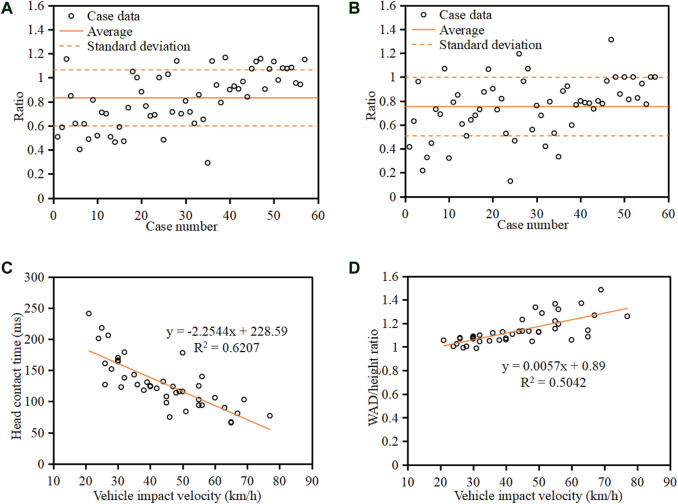
Ratio of head linear contact velocity in km/h **(** **A** **)** and angular contact velocity in rad/s **(** **B** **)** to vehicle impact velocity in km/h and relationships between pedestrian head contact time **(** **C** **)** and WAD/height ratio **(** **D** **)** and vehicle impact velocity for the bonnet-type (sedan and SUV) cars.

A further analysis was conducted to understand the relationship between pedestrian head-to-vehicle contact boundary condition and vehicle impact velocity. Figures 11A,B show the ratio of pedestrian head linear and angular contact velocity to vehicle impact velocity for each reconstructed case, together with the average value and standard deviation (SD) range for the sample. It should be noted that the ratio of head angular contact velocity to vehicle impact velocity is in the unit of rad/s over km/h. Pedestrian head linear contact velocity is on average 83% (SD range: 60%–106%) of the vehicle impact velocity, while the head angular contact velocity (in rad/s) is on average 75% (SD range: 50%–100%) of the vehicle impact velocity in km/h. Figures 11C,D show the relationships between pedestrian head contact time and WAD/height ratio and vehicle impact velocity for the bonnet-type (sedan and SUV) cars, where the cases involving MPVs were not included as their flatter front end can significantly affect pedestrian kinematics. Clearly, negative and positive linear correlations are observed for the relationships between pedestrian head contact time and WAD/height ratio and vehicle impact velocity, respectively.

### Pedestrian AIS3+ Head Injury Risk

**FIGURE 12:**
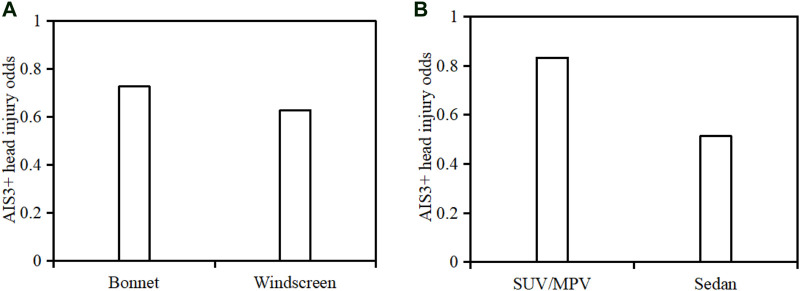
Comparison of AIS3+ head injury odds between bonnet and windscreen impacts **(** **A** **)** and between sedan and SUV/MPV crashes **(** **B** **)**.

Figure 12 compares the AIS3+ head injury odds (the number of cases with AIS3+ injuries over the number of all cases) between bonnet and windscreen impacts as well as between sedan and SUV/MPV crashes. Clearly, the AIS3+ head injury odds are higher for impacts with bonnet (0.73) than for those with windscreen (0.63) and also higher for SUV/MPV crashes (0.83) than for sedan cases (0.51).

**FIGURE 13:**
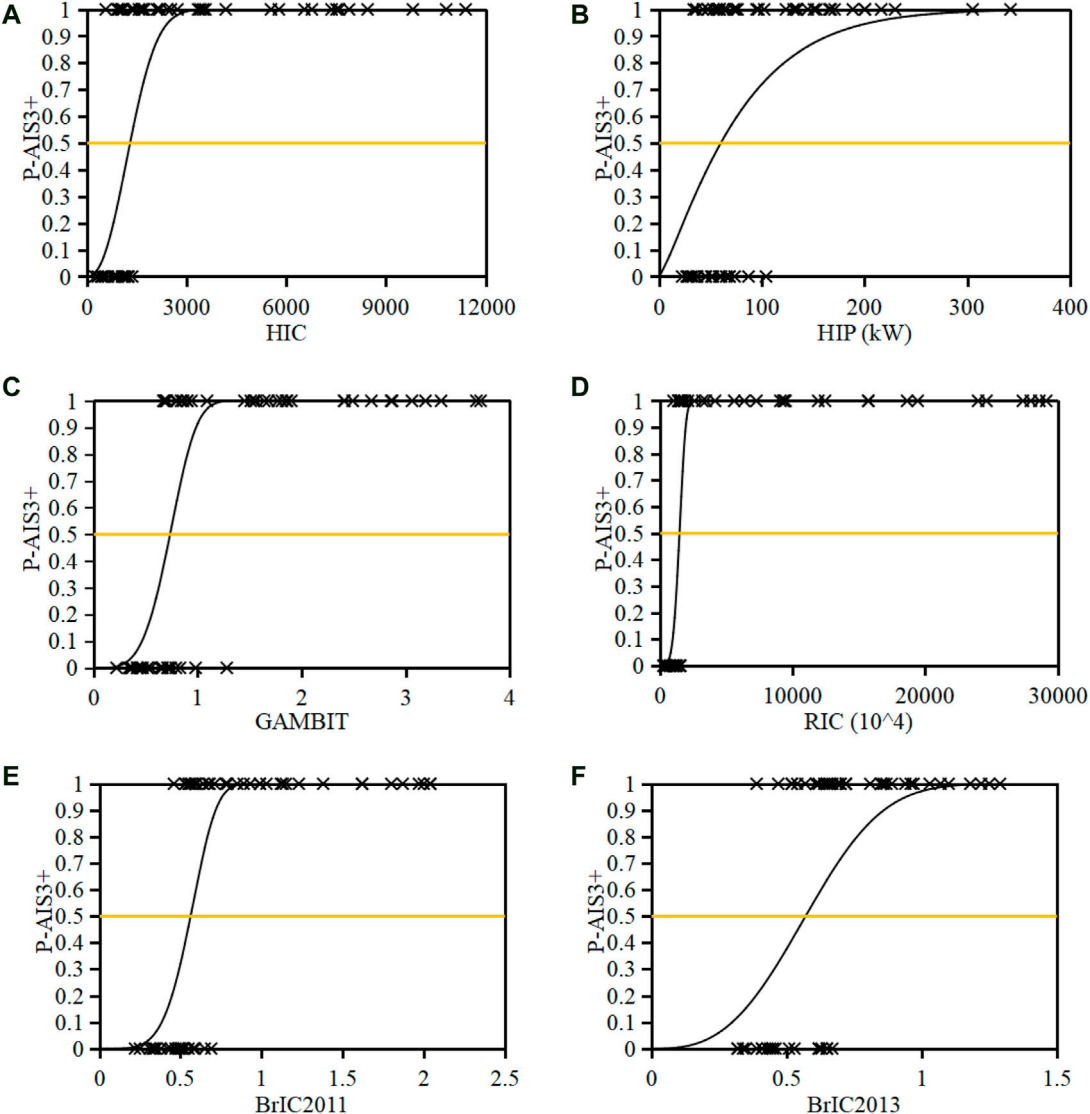
Pedestrian head AIS3+ injury risk as functions of HIC **(** **A** **)**, HIP **(** **B** **)**, GAMBIT **(** **C** **)**, RIC **(** **D** **)**, and BrIC **(** **E**–**F** **)**.

Supplementary Appendix Figure S1 shows the predicted HIC, HIP, GAMBIT, RIC, and BrIC for each case, where the magnitudes of the AIS1-2 group are obviously lower than that of the AIS3+ group for all criteria. Figure 13 shows pedestrian AIS3+ head injury risk curves as functions of HIC, HIP, GAMBIT, RIC, and BrIC estimated using the Weibull model and the data shown in Supplementary Appendix Figure S1. Table 1 presents the coefficients *a* and *b* in Eq. 2 and the AUC value for each Weibull model, where the AUC values are generally higher than 75% and can prove the good predictive capability of the Weibull models. Taking the 50% AIS3+ head injury risk as the cut line, the thresholds are 1,300 for HIC, 60 kW for HIP, 0.74 for GAMBIT, 1,470 × 10^4^ for RIC, 0.56 for BrIC2011, and 0.57 for BrIC2013. The predictions of the current work were compared with previous studies focusing on the thresholds for 50% risk of different head injuries in Table 2.

**TABLE 1:** Weibull model results for HIC, HIP, GAMBIT, RIC, and BrIC.

Predictor	HIC	HIP	GAMBIT	RIC	BrIC2011	BrIC2013
a	1,519.013	81.359	0.803	1,593.119	0.606	0.647
b	2.321	1.188	4.110	4.343	4.963	2.930
AUC	84.2%	64.8%	84.2%	84.2%	84.2%	73.7%

**TABLE 2:** Thresholds for 50% risk of head injuries from different studies.

Study	Head injury	HIC	HIP (kW)	GAMBIT	RIC	BrIC2011	BrIC2013
Current work	AIS3+	1,300	60	0.74	1,470 × 10^4^	0.56	0.57
Peng et al. (2013)	AIS3+	1,442	—	—	—	—	—
Mertz et al. (1996)	Skull fracture	1,400–1,600	—	—	—	—	—
Mertz et al. (2003)	Skull fracture	1,450	—	—	—	—	—
Marjoux et al. (2008)	Skull fracture	667	38	—	—	—	—
	Severe neurological injury	1,032	48	—	—	—	—
	Subdural hematoma	1,429	55	—	—	—	—
Newman (1986)	AIS3+	—	—	1.0	—	—	—
Kimpara and Iwamoto (2012)	Mild TBI	—	—	—	1,030 × 10^4^	—	—
Takhounts et al. (2011)	AIS3+ TBI	—	—	—	—	1.0	—
Takhounts et al. (2013)	AIS3+ TBI	—	—	—	—	—	0.95/0.87

**FIGURE 14:**
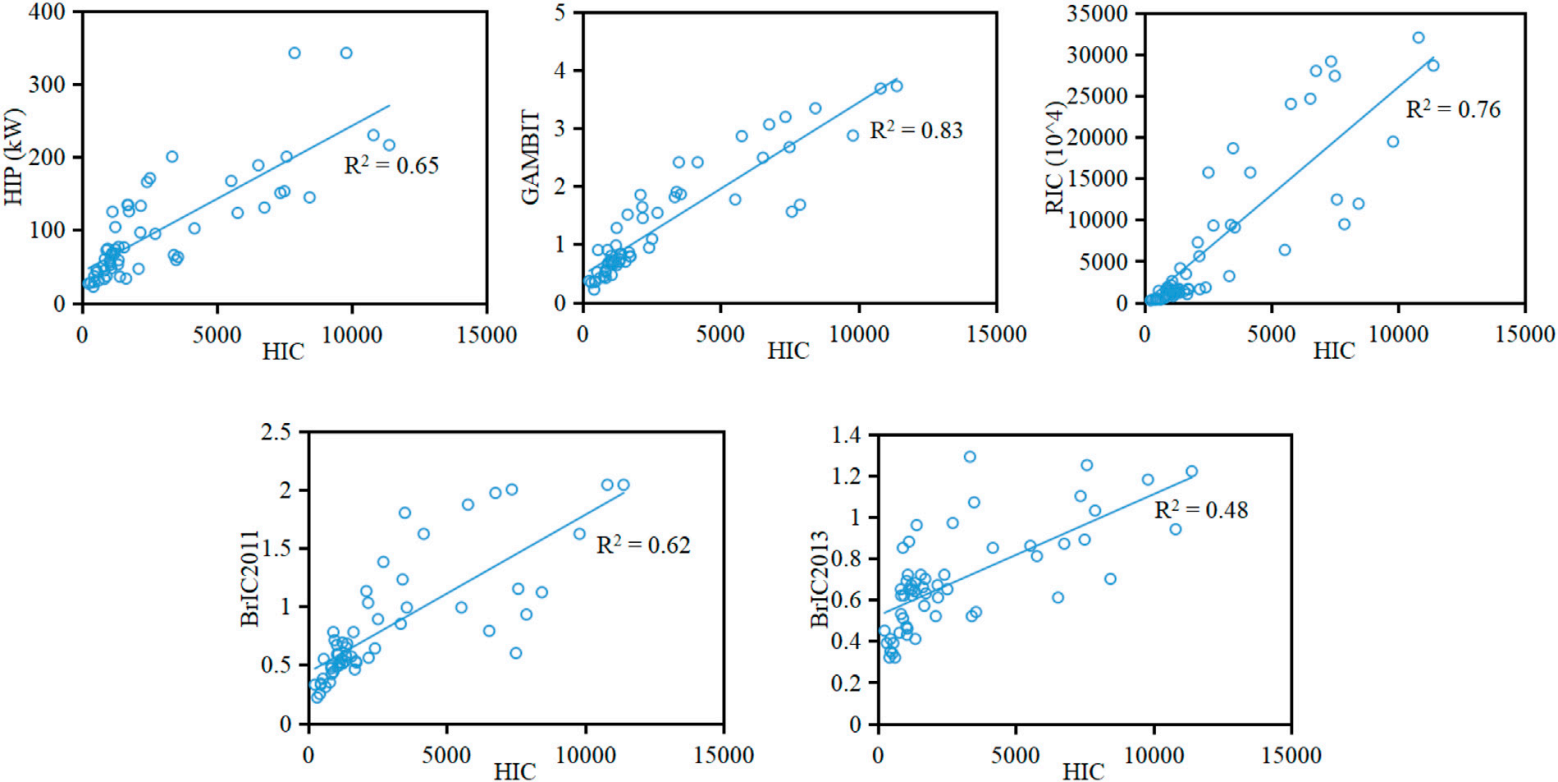
Linear regression between HIC and HIP, GAMBIT, RIC, and BrIC.

The relationships between HIC and other kinematic-based criteria analyzed using linear regression are shown in Figure 14, where the GAMBIT and RIC show a higher linear correlation to the HIC than to the HIP and BrIC, and the linear fit between BrIC2013 and HIC has the lowest *R* ^2^ value of all the correlations.

## Discussion

### Reference for Setup of Pedestrian Head-to-Vehicle Impact Boundary Condition

The current work presents the data of Chinese adult pedestrian head-to-vehicle contact boundary condition from kinematic reconstruction of real-world collisions. The results show that head-to-windscreen contacts (61.4%) are dominant in pedestrian collisions of the analysis sample, and head contact points cover the WAD range 1.5–2.3 m with 74% of cases in the range 1.6–2.0 m and an average value of 1.84 m (Figure 7B). The observed average WAD value from the current work is similar to that reported by Nie et al. (2014) based on the analysis of 21 cases from Changsha city of China (1.82 m), but distinguished with that from Germany data (Kiuchi et al., 2015) and the mixed data of China and Germany (Peng et al., 2013), where the average WAD is over 2.0 m. The smaller WAD of Chinese pedestrians compared to that of Germans is mainly due to the shorter height of the Chinese population. These findings may suggest that the windscreen is an important part for pedestrian head protection for vehicle safety assessment in the Chinese market and that a closer focus should be paid on the area within a WAD of 1.6–2.0 m, which is the main overlap range of the bonnet and windscreen (Figure 7), and legislative regulations should consider local characteristics. However, in the current C-IASI and C-NCAP, the adult head impactor test area for pedestrian protection mainly focuses on the WAD range 1.7–2.1 m (C-IASI, 2017; C-NCAP, 2020), so about 30% of adult head contacts on the vehicle observed in the current study are not included (Figure 7).

For head linear contact velocity, the current C-NCAP and C-IASI use 40 km/h in the subsystem tests (C-IASI, 2017; C-NCAP, 2020). However, as observed in the current work (Figure 8A), this value can cover only 70% cases of the current sample. If coverage of 80% of cases is considered (regarded as the cut line for legislation similar to the EEVC, 2002), the head linear contact velocity should be 45 km/h as observed here (Figure 8A). This implies that the current C-NCAP and C-IASI may overestimate the safety performance of vehicles in rear-world collisions given the recognized positive correlation between head linear impact velocity and pedestrian injury risk (Wang et al., 2020). For head angular contact velocity, the reconstruction results show that more than 67% of cases have a head angular contact velocity in the range 20–40 rad/s (Figure 8B). If coverage of 80% of cases is similarly considered, the head angular contact velocity is 40 rad/s (Figure 8B). The simulation data of the current work also indicate that the MPVs and SUVs could lead to a higher angular head contact velocity for pedestrians compared with sedans (Figure 10B); this trend is similar to that of cadaver and dummy tests (Kerrigan et al., 2009) and may be largely due to the stronger upper body rotation caused by the higher bonnet leading edge of MPVs and SUVs. Many previous studies have indicated that head rotational motion has a significant influence on brain response and injury risk (Gennarelli et al., 1971; Rowson et al., 2011; Takhounts et al., 2011 and 2013). However, all current pedestrian safety regulations have not considered this impact boundary parameter in the subsystem tests. The above data could provide reference for future pedestrian safety regulations to consider the effect of head angular motion. It should be noted that the EEVC proposed the impact velocity for impactor tests according to vehicle impact velocity rather than the contact velocity of the corresponding body part, while the head impact boundary condition suggested by the current work was based on the observed head-to-vehicle contact data. On the other hand, the big gap between head-to-vehicle contact velocity and vehicle impact speed (Figures 11A,B) may imply that the impact velocity for head impactor tests directly sourced from the head-to-vehicle contact boundary condition might be more reasonable. Furthermore, the ratio of respectively 83% (SD range: 60–106%) and 75% (SD range: 50–100%) for pedestrian head linear (km/h) and angular (rad/s) contact velocity to vehicle impact velocity (km/h) observed in the current work (Figures 11A,B) could provide reference for estimation of pedestrian head-to-vehicle contact velocity from vehicle initial impact speed.

**FIGURE 15:**
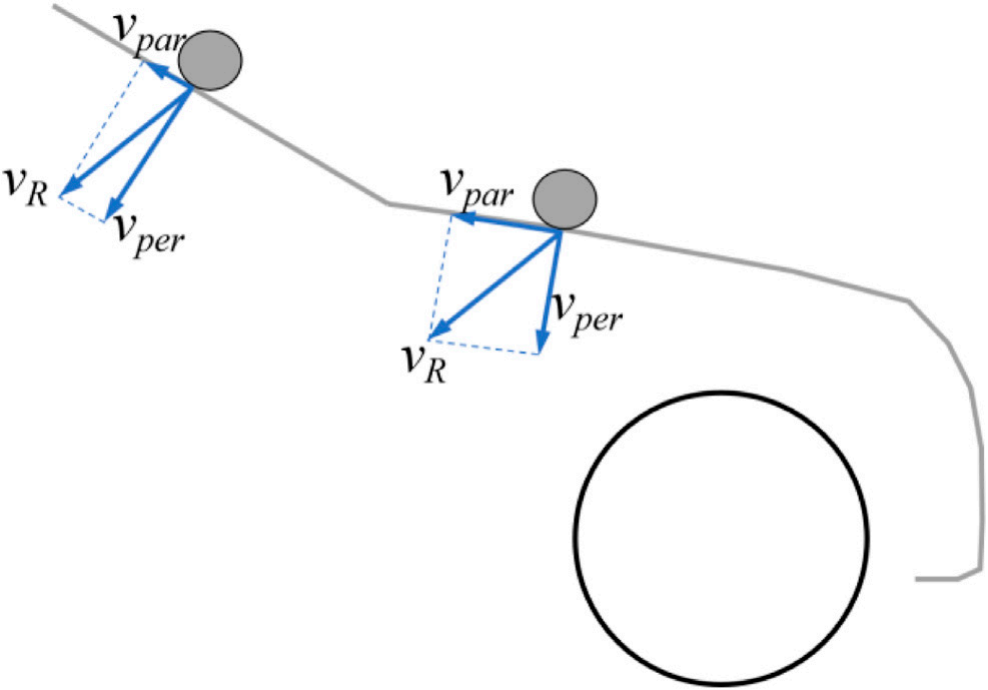
Decomposition of head contact velocity in bonnet and windscreen impacts.

The results indicate that 70% of cases have a head contact angle in the range 40–70°, and the average value is 53° (Figure 8D). This finding is similar to that of Nie et al. (2014), but the average value is 12° less than the 65° defined in C-NCAP and C-IASI. Given the dramatic difference in the inclination angle between windscreen (23–55°, median = 30°) and bonnet (8–37°, median = 13°) (Li et al., 2017a), the same head linear contact velocity at a certain incident angle to the bonnet and windscreen will lead to an obviously different perpendicular impact velocity to the head (Figure 15), which is the principal component for the head impact load. Moreover, Figure 9D indicates that the head contact angle for the cases with bonnet impacts (average = 60°) is significantly higher than that for windscreen impacts (average = 48°). These findings may suggest that the current C-NCAP and C-IASI might need to reduce the adult head contact angle in the test procedures and to distinguish the bonnet and windscreen.

Pedestrian head contact time and WAD (determining the contact location) are important factors for pressure and structure control in the development of pedestrian airbag (Lim et al., 2015). The kinematic reconstruction data show that the head contact time is mainly (77%) in the range of 50–140 ms (Figure 8C), which is affected by vehicle type with a significantly earlier head contact time in SUV/MPV crashes than in collisions with sedans (Figure 10C) and also affected by vehicle impact velocity by a negative linear correlation (Figure 11C). The results also show that the WAD is influenced by pedestrian height and vehicle impact velocity, where a positive linear correlation is observed for the relationship between pedestrian WAD/height ratio and vehicle impact velocity (Figure 11D). These findings could provide evidence for estimation of pedestrian head contact time and location from accident scenarios.

### Reference for Estimation of Pedestrian Head Injury Risk

The predicted thresholds of HIC, HIP, GAMBIT, RIC, BrIC2011, and BrIC2013 for a 50% probability of AIS3+ head injury risk are 1,300, 60 kW, 0.74, 1,470 × 10^4^, 0.56, and 0.57, respectively (Figure 13). The comparisons in Table 2 indicate that the HIC of 1,300 for 50% AIS3+ head injury risk observed in the current work is similar to that of 1,442 from a previous reconstruction study of pedestrian collisions (Peng et al., 2013), that of 1,450 for 50% of skull fracture reported by Mertz et al. (2003) in the analysis of the data from Injury Assessment Reference Values, and that of 1,429 for 50% subdural hematoma risk predicted from reconstructions of mixed data of motorcyclist, footballer, and pedestrian accidents using an isolated human body head FE model (Marjoux et al., 2008). However, the HIC of 1,300 is much higher than that of 667 for a 50% skull fracture risk and 1,032 for a 50% severe neurological injury risk observed in the study of Marjoux et al. (2008). The HIP of 60 kW for 50% AIS3+ head injury risk predicted here is higher than that of 38 kW for 50% skull fracture risk and 48 kW for 50% severe neurological injury risk, but similar to that of 55 kW for 50% subdural hematoma risk observed from Marjoux et al. (2008). The GAMBIT of 0.74 for 50% AIS3+ head injury risk predicted by the current work is a little lower than that of 1.0 proposed by Newman (1986). The RIC of 1,470 × 10^4^ for a 50% AIS3+ head injury risk estimated from current reconstruction data is obviously higher than that of 1,030 × 10^4^ for 50% mild TBI (AIS2) (Kimpara and Iwamoto, 2012), but no threshold is available in previous studies for AIS3+ head injuries. The predicted thresholds of BrIC2011 and BrIC2011 for a 50% AIS3+ head injury risk are much lower than those reported in previous studies from reconstructions of dummy impact tests of vehicle occupants (Takhounts et al., 2011 and 2013).

The above analysis suggests that the thresholds for the same injury level and criterion may vary from study methods (isolated FE human body head modeling, multi-body full body modeling, tests of ATDs, etc.) and data sample (pedestrian, motorcyclist, footballer, and occupant accidents or mixed data). Generally, the thresholds of HIC, HIP GAMBIT, and RIC for a 50% AIS3+ head injury risk predicted from current reconstruction data are plausible when compared to previous studies. However, the thresholds for BrIC2011 and BrIC2011 show a big gap with the data from Takhounts et al. (2011) and Takhounts et al. (2013). This difference might be due to the fact that the values of BrIC should be used in conjunction with the injury assessment device it is measured with as claimed by Takhounts et al. (2011) and Takhounts et al. (2013). In Euro-NCAP, C-NCAP, and C-IASI, the HIC of 650 and 1,700 are regarded as the thresholds for “ADEQUATE” and “POOR” performance of vehicle safety, respectively (C-IASI, 2017; C-NCAP, 2020; Euro-NCAP 2020). These cut lines were defined based on a specific risk of skull fracture observed from studies of biomechanics. Similarly, the predicted head injury risk curves of the current work could provide reference for this, considering various kinematic-based criteria HIC, HIP, GAMBIT, RIC, and BrIC.

HIC is currently used in NCAPs for estimation of pedestrian injury risk, but other kinematic-based criteria could be potential supplements for the evaluation of pedestrian head injuries given the limitation of HIC. The linear correlation analysis between HIC and other kinematic-based criteria (Figure 14) indicates that HIC provides a higher correlation (*R* ^2^ > 0.7) to GAMBIT and RIC than to HIP and BrIC. This is mainly due to the fact that the physical quantities considered in the formulations of HIC, GAMBIT, and RIC are head acceleration (linear and/or angular) (Supplementary Appendix Information S1), while HIP and BrIC include head angular velocity; particularly, BrIC2013 considers head angular velocity only and has the lowest *R* ^2^ value (0.48) to HIC. This finding may suggest that HIC could generally represent the kinematic-based criteria considering acceleration only, such as the GAMBIT and RIC, but could not represent the criteria which include head angular velocity which could be considered as potential supplements to HIC for evaluation of pedestrian head injuries. On the other hand, the AUC values indicate that the predictive capability of the injury curves developed based on HIP (AUC = 64.8%) and BrIC2013 (AUC = 73.7%) is lower than that based on other kinematic-based criteria (AUC = 84.2%) for the current analysis sample. The above findings may suggest that GAMBIT, RIC, and BrIC2011 could be considered as the potential supplements of HIC for evaluation of pedestrian head injuries.

### Limitations

There are several limitations in the current study. Firstly, there are some uncertainties in the information of accident data, especially the estimated vehicle impact speed and initial posture of the pedestrian, which could have a significant influence on the reconstruction results, but the optimization process can help to reduce the estimation error. Secondly, the human body model for an individual case was scaled from the 50th% adult male model, where characteristics of joints and stature could only be approximated and the age effect on the mechanical properties of human body was not considered in the model. Thirdly, stiffness curves of vehicle models were defined according to the available rating results of the vehicle models in subsystem tests and the corresponding stiffness curves published in previous studies since it is not able to access the test data for the exact vehicle model given the public unavailability of stiffness curves for vehicle models. This could lead to some uncertainties to the predicted magnitudes of head injury criteria. Although there are many shortcomings for this approach, this is one of the reasonable solutions for stiffness assumption and widely used in pedestrian crash reconstructions (Nie et al., 2014; Peng et al., 2012; Wu et al., 2021), and the good match in the AIS level between the predictions and real-world accident data (Figure 5) could support the reliability of this approach. Finally, the parameters such as contact friction coefficients were defined according to the literature, which also led to some uncertainties in the modeling. However, there are inherent drawbacks in the reconstruction study of real-world collisions (Huang er al., 2018; Nie et al., 2014; Penr et al., 2013), and further improvements in the availability of data information for accident scenarios, mechanical properties of the human body and vehicles, and the modeling approach are needed to overcome these difficulties.

## Conclusion

This study is the first attempt at understanding the characteristics of Chinese pedestrian head-to-vehicle contact boundary condition considering rotational contact velocity and head injury risk as functions of the kinematic-based criteria HIC, HIP, GAMBIT, RIC, and BrIC from reconstructions of real-world collisions using full-body human body models. The findings of the current work could provide realistic reference for evaluation of vehicle safety performance focusing on pedestrian protection, which are summarized as follows:

(1) Head-to-windscreen contacts are dominant in pedestrian collisions of the analysis sample, and head WAD floats from 1.5 to 2.3 m, with a mean value of 1.84 m.
(2) Eighty percent of cases have a head linear contact velocity below 45 km/h or an angular contact velocity less than 40 rad/s; pedestrian head linear contact velocity is on average 83 ± 23% of the vehicle impact velocity, while the head angular contact velocity (in rad/s) is on average 75 ± 25% of the vehicle impact velocity in km/h.
(3) Seventy-seven percent of cases have a head contact time in the range 50–140 ms, and negative and positive linear correlations are observed for the relationship between pedestrian head contact time and WAD/height ratio and vehicle impact velocity, respectively.
(4) Seventy percent of cases have head contact angle floats from 40 to 70°, with an average value of 53°; the pedestrian head contact angles on windscreens (average = 48°) are significantly lower than those on bonnets (average = 60°).
(5) AIS3+ head injury risk curves as functions of kinematic-based criteria HIC, HIP, GAMBIT, RIC, BrIC2011, and BrIC2013 were built, where the thresholds for a 50% probability of AIS3+ head injury risk are 1,300, 60 kW, 0.74, 1,470 × 10^4^, 0.56, and 0.57, respectively.
(6) HIC provides a high linear correlation to the kinematic-based criteria considering acceleration only (GAMBIT and RIC), but a lower linear correlation to the criteria which include head angular velocity (HIP and BrIC).

## Data Availability

The original contributions presented in the study are included in the article/Supplementary Material, further inquiries can be directed to the corresponding authors.
