# Supplementary material for: Realistic Reference for Evaluation of Vehicle Safety Focusing on Pedestrian Head Protection Observed From Kinematic Reconstruction of Real-World Collisions
Source: Front Bioeng Biotechnol. 2021 Dec 21;9:768994. doi: 10.3389/fbioe.2021.768994 (PMC8724547; doi:10.3389/fbioe.2021.768994)
Supplement: Supplementary file 1 [file DataSheet1.docx]

# Appendix A

**Table A1.** General information of the real world cases.

| Case No. | Vehicle information | | | Pedestrian information | | |
| --- | --- | --- | --- | --- | --- | --- |
|  | Brand | Type | Speed (km/h) | Gender | Height (cm) | Head MAIS |
| 1 | MG | Sedan | 65 | M | 166 | 5 |
| 2 | Hover | MPV | 46 | F | 154 | 3 |
| 3 | Honda | Sedan | 26 | F | 163 | 5 |
| 4 | BMW | Sedan | 46 | F | 153 | 5 |
| 5 | Trumpchi | Sedan | 55 | M | 181 | 5 |
| 6 | Honda | Sedan | 47 | F | 155 | 3 |
| 7 | Honda | Sedan | 26 | M | 158 | 5 |
| 8 | Changan | Sedan | 45 | F | 153 | 4 |
| 9 | Changan | MPV | 43 | F | 159 | 6 |
| 10 | VW | Sedan | 56 | M | 173 | 4 |
| 11 | Changan | MPV | 38 | F | 151 | 5 |
| 12 | FAW | MPV | 40 | M | 167 | 5 |
| 13 | Wuling | MPV | 61 | F | 150 | 3 |
| 14 | Geely | Sedan | 69 | F | 156 | 5 |
| 15 | BYD | Sedan | 39 | M | 178 | 3 |
| 16 | Changan | MPV | 53 | F | 158 | 2 |
| 17 | Hyundai | MPV | 48 | F | 151 | 5 |
| 18 | Changan | MPV | 40 | M | 176 | 5 |
| 19 | Changan | MPV | 46 | F | 155 | 6 |
| 20 | Dongfeng | MPV | 51 | F | 148 | 5 |
| 21 | Changan | Sedan | 55 | F | 171 | 6 |
| 22 | Changan | MPV | 44 | M | 160 | 4 |
| 23 | Toyota | Sedan | 55 | M | 160 | 4 |
| 24 | KIA | Sedan | 31 | M | 179 | 6 |
| 25 | Changan | Sedan | 60 | M | 170 | 5 |
| 26 | Changan | MPV | 36 | F | 145 | 4 |
| 27 | Changan | MPV | 28 | M | 156 | 6 |
| 28 | Changan | MPV | 50 | M | 158 | 4 |
| 29 | Changan | Sedan | 50 | M | 170 | 4 |
| 30 | Changan | Sedan | 67 | F | 153 | 6 |
| 31 | Volvo | Sedan | 56 | F | 161 | 2 |
| 32 | VW | Sedan | 50 | M | 171 | 2 |
| 33 | Lexus | Sedan | 63 | F | 162 | 5 |
| 34 | Lexus | Sedan | 49 | F | 140 | 6 |
| 35 | Foday | SUV | 24 | F | 165 | 6 |
| 36 | Changan | Sedan | 51 | M | 166 | 6 |
| 37 | KIA | SUV | 65 | F | 165 | 6 |
| 38 | Changan | Sedan | 77 | M | 180 | 6 |
| 39 | VW | Sedan | 30 | F | 165 | 1 |
| 40 | Toyota | Sedan | 30 | M | 170 | 1 |
| 41 | Honda | Sedan | 28 | F | 165 | 1 |
| 42 | VW | Sedan | 32 | M | 175 | 1 |
| 43 | BYD | Sedan | 30 | M | 175 | 1 |
| 44 | Nissan | Sedan | 25 | M | 170 | 1 |
| 45 | Audi | Sedan | 27 | M | 165 | 1 |
| 46 | VW | Sedan | 30 | M | 175 | 1 |
| 47 | VW | Sedan | 32 | M | 170 | 1 |
| 48 | Buick | Sedan | 21 | M | 175 | 1 |
| 49 | Toyota | Sedan | 42 | F | 165 | 2 |
| 50 | Honda | Sedan | 45 | M | 170 | 2 |
| 51 | VW | SUV | 48 | F | 155 | 2 |
| 52 | VW | Sedan | 38 | F | 160 | 2 |
| 53 | VW | Sedan | 40 | M | 165 | 2 |
| 54 | Hyundai | Sedan | 36 | M | 180 | 2 |
| 55 | Honda | Sedan | 44 | M | 170 | 2 |
| 56 | Chery | SUV | 35 | F | 155 | 2 |
| 57 | VW | Sedan | 40 | M | 170 | 2 |

# Appendix B

Formulations for HIC, HIP, GAMBIT, RIC, and BrIC are given as below:

$HIC=\left\{ \left[ \frac{1}{t_{2}-t_{1}}\int_{t_{1}}^{t_{2}} a_{t}dt \right]^{2.5}\left( t_{2}-t_{1} \right) \right\}_{max}$ (B1)

$HIP=C_{1}a_{x}\int a_{x}dt+C_{2}a_{y}\int a_{y}dt+C_{3}a_{z}\int a_{z}dt+C_{4}\partial_{x}\int\partial_{x}dt+C_{5}\partial_{y}\int\partial_{y}dt+C_{6}\partial_{z}\int\partial_{z}dt$ (B2)

$GAMBIT=\left[ \left( \frac{a_{max}}{a_{cr}} \right)^{2}+\left( \frac{\partial_{max}}{\partial_{cr}} \right)^{2} \right]^{\frac{1}{2}}$ (B3)

$RIC=\left\{ \left[ \frac{1}{t_{2}-t_{1}}\int_{t_{1}}^{t_{2}} \alpha_{t}dt \right]^{2.5}\left( t_{2}-t_{1} \right) \right\}_{max}$ (B4)

$BrIC2011=\frac{\omega_{max}}{\omega_{cr}}+\frac{\alpha_{max}}{\alpha_{cr}}$ (B5)

$BrIC2013=\left[ \left( \frac{\omega_{x}}{\omega_{xc}} \right)^{2}+\left( \frac{\omega_{y}}{\omega_{yc}} \right)^{2}+\left( \frac{\omega_{z}}{\omega_{zc}} \right)^{2} \right]^{\frac{1}{2}}$ (B6)

where in Eq. (B1) *a_t_* is head linear acceleration, *t_2_-t_1_* is 15ms (Versace, 1971); in Eq. (B2) *C_i_* are the mass and moments of inertia for the human head, while *a_x_*, *a_y_*, *a_z_* are head linear acceleration components and *∂_x_*, *∂_y_*, *∂_z_* are head rotational acceleration components (Newman et al., 2000); in Eq. (B3) *a_max_* and *∂_max_* are the maximum linear and rotational accelerations of the head respectively, while *a_cr_* and *∂_cr_* are critical linear and rotational acceleration defined as 250g and 25000rad/s^2^, respectively (Newman, 1986); in Eq. (B4) *α_t_* is head rotational acceleration and *t_2_-t_1_* is 36ms (Kimpara and Iwamoto, 2012); in Eq. (B5) *ω_max_* and *α_max_* are the maximum value of head rotational velocity and acceleration respectively, while *ω_cr_* and *α_cr_* are the critical rotational velocity and acceleration defined as 140rad/s and 25000rad/s^2^ (Takhounts et al., 2011); in Eq. (B6) *ω_x_*, *ω_y_*, *ω_z_* are head rotational velocity components and *ω_xc_*, *ω_yc_*, *ω_zc_* are the corresponding critical values defined as 66.25rad/s, 56.45rad/s and 42.87rad/s, respectively (Takhounts et al., 2013).

# Appendix C


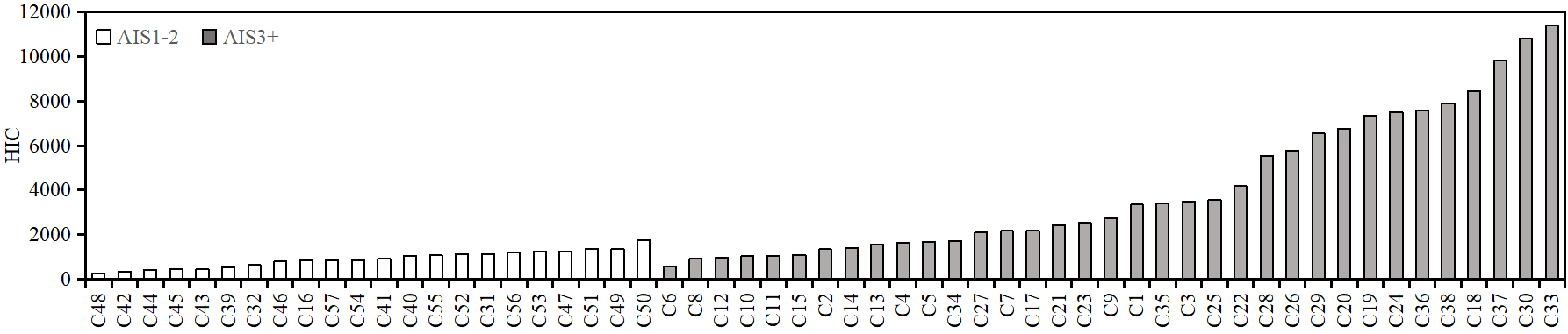


(a)


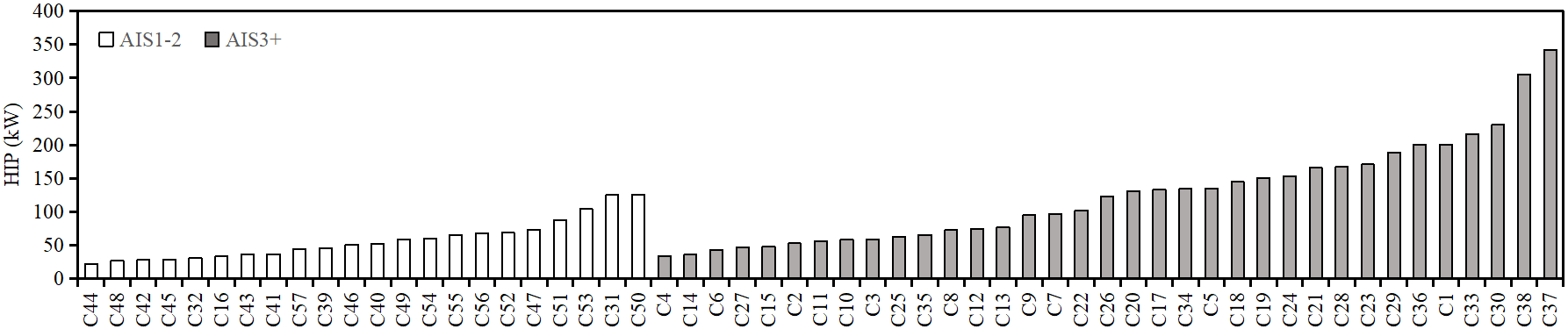


(b)


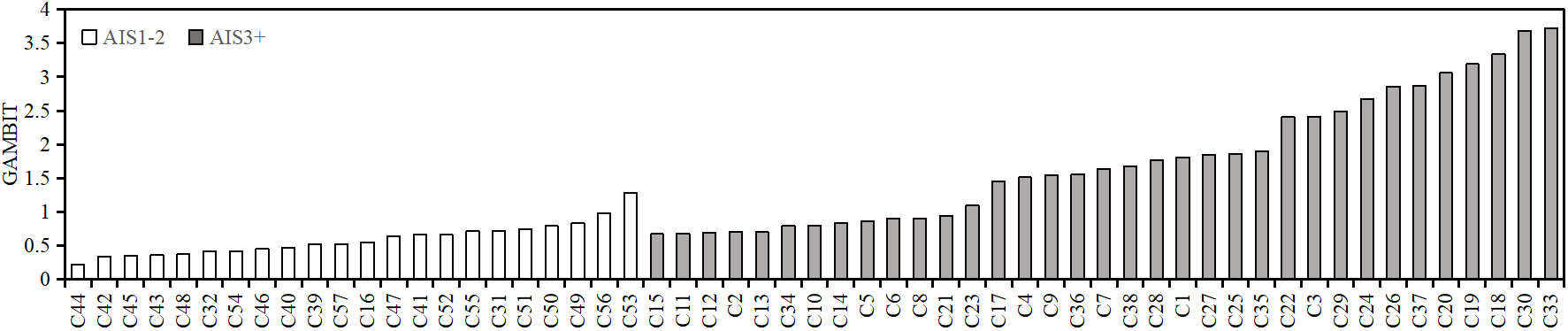


(c)


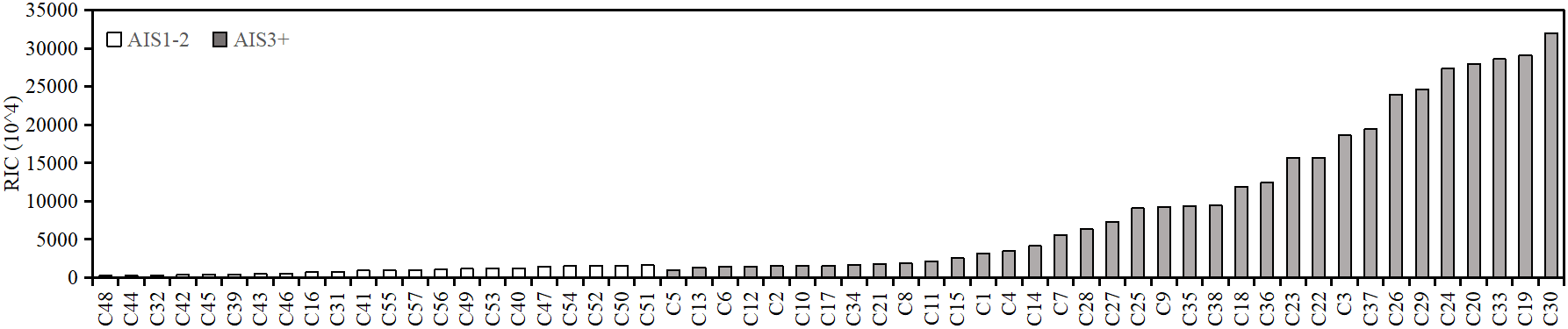


(d)


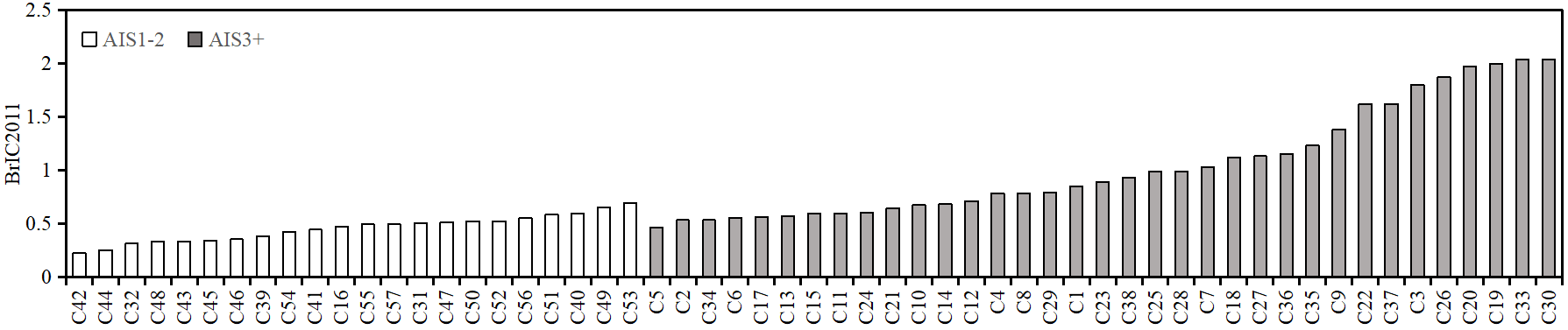


(e)


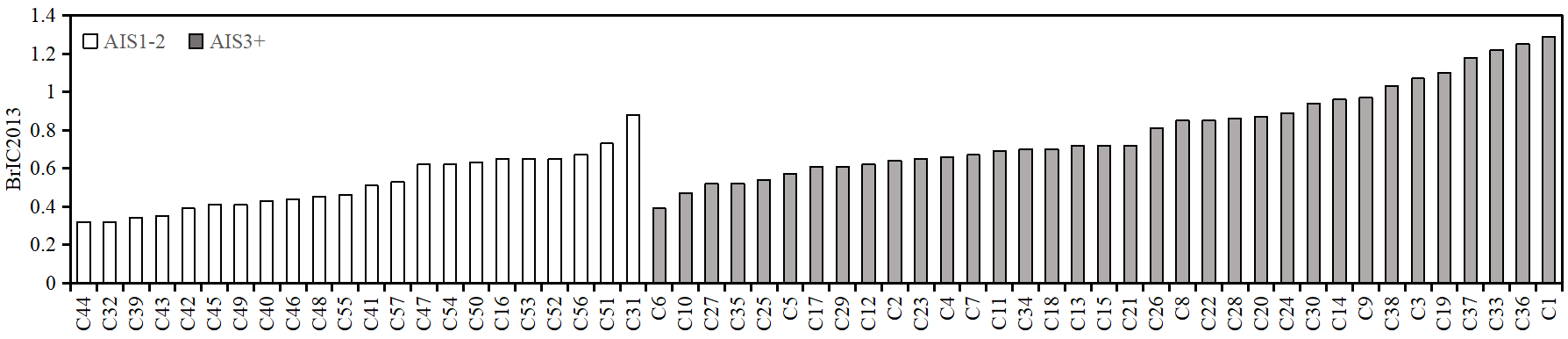


(f)

**Fig. C1.** Predicted HIC (a), HIP (b), GAMBIT (c), RIC (d), and BrIC (e-f) values for each case.
